# Training rhesus macaques to take daily oral antiretroviral therapy for preclinical evaluation of HIV prevention and treatment strategies

**DOI:** 10.1371/journal.pone.0225146

**Published:** 2019-11-15

**Authors:** Michele B. Daly, April M. Clayton, Susan Ruone, James Mitchell, Chuong Dinh, Angela Holder, Julian Jolly, J. Gerardo García-Lerma, James L. Weed

**Affiliations:** 1 Laboratory Branch, Division of HIV/AIDS Prevention, Centers for Disease Control and Prevention, Atlanta, Georgia, United States of America; 2 Comparative Medicine Branch, Division of Scientific Resources, National Center for Emerging and Zoonotic Infectious Diseases, Centers for Disease Control and Prevention, Atlanta, Georgia, United States of America; Tulane National Primate Research Center, UNITED STATES

## Abstract

**Background:**

Macaque models of simian or simian/human immunodeficiency virus (SIV or SHIV) infection are critical for the evaluation of antiretroviral (ARV)-based HIV treatment and prevention strategies. However, modelling human oral ARV administration is logistically challenging and fraught by limited adherence. Here, we developed a protocol for administering daily oral doses of ARVs to macaques with a high rate of compliance.

**Methods:**

Parameters of positive reinforcement training (PRT), behavioral responses and optimal drug delivery foods were defined in 7 male rhesus macaques (*Macaca mulatta*). Animals were trained to sit in a specified cage location prior to receiving ARVs, emtricitabine (FTC) and tenofovir alafenamide (TAF), in a blended food mixture, which was followed immediately with a juice chaser. Consistency of daily oral adherence was evaluated in 4 trained macaques receiving clinically equivalent doses of FTC and TAF (20 and 1.5 mg/kg, respectively) in a short-term (1 month) and an extended (6 month) trial. Adherence was monitored using medication diaries and by quantifying intracellular FTC-triphosphate (FTC-TP) and tenofovir-diphosphate (TFV-DP) concentrations in peripheral mononuclear blood cells (PBMCs).

**Results:**

Trained macaques quickly and consistently took daily oral ARVs for 1 month with an average 99.8% observed adherence. Intracellular concentrations of TFV-DP (median = 845.8 fmol/million cells [range, 620.8–1031.3]) and FTC-TP (median = 367.0 fmol/million cells [range, 289.5–413.5) in PBMCs were consistent with high adherence. Extended treatment with select subjects yielded similar observations for three months (99.5% adherence, 352/356 complete doses taken), although a sudden drop in adherence was observed after splenic biopsy surgery.

**Conclusions:**

We demonstrate that trained macaques reliably adhere to a daily oral ARV regimen, although unexpected adherence issues are possible. Our approach, using clinical doses of oral FTC and TAF daily, further refines macaque models of HIV treatment and prevention by mimicking the human route and timing of ARV administration.

## Introduction

Over thirty years after its discovery, human immunodeficiency virus (HIV) remains a significant public health concern with an estimated 36.7 million people infected worldwide [1]. Antiretroviral (ARV) therapy has substantially improved the longevity of persons living with HIV. However, ARV treatment is not curative, and patients must strictly adhere to a daily oral regimen to prevent viral rebound and progression to acquired immunodeficiency syndrome (AIDS).

Macaque models of HIV infection have contributed significantly to our understanding of HIV pathogenesis, immune response, prophylaxis and treatment (reviewed in [2]). The anatomical and physiological similarities between macaques and humans, as well as the homology between HIV and simian or simian/human immunodeficiency viruses (SIV or SHIV) make these models attractive for evaluation of novel HIV prevention modalities, treatment, and cure strategies. Strict experimental parameters can be employed when using macaque models, which can eliminate confounding variables seen in clinical trials, such as ARV adherence [3]. In efforts to provide data with the highest preclinical and translational relevance, animal models are continuously refined to ensure that human infection and treatment is mirrored as closely as possible. Recent macaque model refinements include repeat rectal and vaginal SHIV exposures to mimic high risk sexual behavior [4, 5], co-infection with sexually transmitted infections [6, 7] and the use of human equivalent doses of ARVs to model HIV treatment and pre-exposure or post-exposure prophylaxis modalities (PrEP or PEP) [8, 9].

HIV treatment is almost exclusively given as a single co-formulated daily pill, which has decreased pill burden and increased patient adherence [10]. However, modeling daily oral ARV administration poses unique obstacles for macaque studies because oral treatments, which are often unappealing in smell and/or taste, are not easily masked in food. Secondly, an input of time is required for animal training to ensure that oral doses are eaten reliably, and lastly, observation is necessary to confirm individual dose consumption [11]. These logistical challenges have resulted in many macaque studies administering ARVs via daily subcutaneous injections or nondaily by oral gavage. Drug pharmacokinetics can be affected by route of drug administration and dose timing resulting from variations in adsorption, bioavailability, tissue distribution and elimination [12]. Importantly, macaque models utilizing subcutaneous or non-daily oral ARVs may not fully inform on oral biodistribution and efficacy compared to daily oral regimens [8, 13–16].

Positive reinforcement training (PRT) uses incentives, or reinforcers, as motivation for performing a desired behavior. Behavioral research of captive animal food preferences have detailed how to choose effective reinforcers to improve training efficacy, husbandry and animal welfare [17]. Specifically, nonhuman primate researchers have experimentally determined primate food valuation and showed that the use of preferred foods increases engagement during training sessions [18, 19]. In this study, preferred foods were determined based on caregiver observations of increased consumption, and these foods were used as reinforcers during PRT. Clinical research in children with feeding disorders have shown that blending a preferred food with a nonpreferred food, such as bitter medications, can limit taste aversion and increase compliance [20]. Another measure to increase oral consumption in children is chasing, where a liquid is given after a nonpreferred food item to discourage packing, or holding in the mouth without swallowing [21]. Rhesus macaques have cheek pouches, where they often pack foodstuffs for later consumption, and chasing may reduce this behavior.

In this study, our goal was to train rhesus macaques to reliably adhere to a daily oral ARV regimen consisting of clinically relevant doses of the nucleoside reverse transcriptase (RT) inhibitor emtricitabine (FTC) and the nucleotide RT inhibitor tenofovir alafenamide (TAF).

SIV/SHIV prevention and treatment studies require stable levels of ARVs to be maintained within a protective threshold, which necessitates highly adherent animals if a daily oral regimen is pursued. To increase the likelihood of daily oral medication compliance we used a combination of tactics including PRT, blending ARVs with preferred foods, and using a chaser after drug administration. Daily adherence was assessed observationally and through weekly analysis of active intracellular ARV levels during a short-term (1 month) and long-term (6 months) trial. These methods have potential use in nonhuman primate studies of drug biodistribution and pharmacodynamics after oral drug administration, as well as models of oral medication adherence in other captive animals and clinical research settings.

## Material and methods

### Ethics statement

All animal procedures were performed according to NIH guidelines and approved by the Institutional Animal Care and Use Committee (IACUC) of the Centers for Disease Control and Prevention (CDC). Macaques were obtained from Worldwide Primates, Inc, and were housed at the CDC under the care of CDC veterinarians in accordance with the Guide for the Care and Use of Laboratory Animals 8th Ed. All procedures were performed under anesthesia (10 mg/kg ketamine or 2–6 mg/kg telazol; intramuscular), and all efforts were made to minimize distress, improve housing conditions, and to provide enrichment opportunities as per our standard operating procedures. For housing, macaques were maintained in Suburban Surgical quad cages that met or exceeded the minimum size requirements as stipulated in the Guide for the Care and Use of Laboratory Animals [cage dimensions (inches): 32 (height) x 26.5 (width) x 33 (depth)]. Steps were taken to reduce animal stress, which included providing enrichment opportunities [e.g. cage features like swings/perches and objects for the macaques to manipulate], an assortment of food selections like fruits, vegetables or seeds, suitable feeding methods (foraging and task-oriented), and humane interactions with caregivers and research staff. All animals have access to clean, fresh water at all times. Commercial diets are specifically formulated to meet vitamin C requirements. Prior to study initiation, compatible macaques are pair-housed. During the study the macaques were separated into single housing (while permitting eye-contact) with a cage divider. These animals are in the continued care of the CDC.

### Macaque training

Seven male rhesus macaques (mean age, 7.4 years [range, 6–9 years]; mean weight 13.4 kg [range, 11.7–15.4 kg]), who had no record or evidence of prior training, were chosen for PRT. Training consisted of operant conditioning procedures, where the animals are required to perform a task prior to receiving feedback, and differential reinforcement was used for successive approximations of behavior [22, 23]. Verbal commands were used to cue desired behavior and, positive reinforcement was given if the correct behavioral response was achieved [24, 25]. Behavioral observations and time spent training the animals were recorded daily.

Primary reinforcers usually fulfill a biological need, and in animal training they are primarily food-based as the inherent value of a treat is motivational [26]. Initially, we used grapes as primary reinforcers, but we transitioned to sweet liquids delivered from a 60-cc catheter-tip syringe in efforts to acclimate the animals to a syringe for potential medication delivery or chasing purposes. Liquid reinforcers included fruit juices (mango, apple, cranberry, pomegranate, peach and passion fruit), diluted strawberry syrup, diluted molasses, vanilla ensure, kefir, and chocolate milk.

Secondary reinforcers gain significance through association with primary reinforcers, and here both verbal praise and a clicker were used. Clickers provide a distinct auditory stimulus cueing the animals that the correct behavior has been achieved [24, 26]. Macaques were acclimated to reinforcer delivery from a syringe over three weeks and were timed from syringe presentation to complete consumption of reinforcer.

Training included two approximation steps, i) shifting location in enclosure, and ii) sitting in a specific location. Initially, in order to deliver liquid reinforcement, the syringe was placed above the food cup at a location which facilitated delivery and training, thus making it easily accessible for animals to approach. Once trained on the syringe delivery method, all fluids were delivered in the same place for all animals, above the food cup opening. Over the course of the study, animals were housed in different caging configurations, which necessitated shifting monkeys from one side of a quad cage to another. Next, the initial location shift was built upon using successive approximation and macaques were trained to sit in the specified location using the verbal command, “sit”. The clicker signaled the correct behavior of the animal sitting and stationing, followed by delivery of positive reinforcement, either grapes or later juice. Liquid was dispensed after the animal approached or put their mouth on the tip and was never dispensed forcefully. Training sessions were limited to 5 minutes each day per animal or shorter if requested behavior was demonstrated sooner or juice was depleted from the syringe. No reinforcers were given until the animals sat. Building a rapport with the animals and training them to sit in a specific spot prior to receiving reinforcer facilitates the transfer of that procedure to alternate staff, who have little or no knowledge of stimulus-response/reinforcer contingencies used to train nonhuman primates.

### Antiretroviral drug stability in food

Human equivalent doses of FTC (MS Hetero Labs Limited, India) and TAF (Laurus, Labs, India) in macaques have been previously established (20 mg/kg and 1.5 mg/kg, respectively) [9, 27]. FTC and TAF powder for a 10 kg macaque (200 mg of FTC and 15 mg TAF) were mixed with 1.2 g of bitterness masking powder (Fagron) and vortexed into 30 cc of juice (peach, mango, grape, apple or Ensure) until homogenous. Drug concentrations in mixtures were measured by high-performance liquid chromatography-tandem mass spectrometry (HPLC-MS/MS, Sciex, Foster City, CA, Shimadzu Scientific, Columbus, MD). For testing of NRTI stability in a solid food matrix, 200 mg of FTC, 15 mg of TAF, and 600 mg of bitterness masking powder were mixed into approximately 1 tablespoon of Nutella^®^ or 1 tablespoon of peanut butter. These mixtures were divided and maintained at room temperature (23°C), -20°C or -80°C for one week prior to testing by HPLC-MS/MS.

A cocktail of methanol containing internal standards was added to food matrices to precipitate proteins. After a brief centrifugation to remove protein precipitates, the supernatant was evaporated to near dryness and resuspended with mobile phase A. An aqueous-acetonitrile mobile-phase gradient was used to elute the drugs from a C18 column (100*1mm, Imtakt, Portland Oregon) and into the analyzer. Drug concentrations were calculated from a standard curve with a range of 0.5–2000 ng/mL using Analyst software. The lower limit of quantification of this assay was 10 ng/mL for both analytes [27].

### Adherence trials

Six macaques from the PRT cohort were enrolled in a 30-day oral FTC/TAF adherence trial and were arbitrarily housed in two separate rooms (Groups A and B). The seventh PRT animal was excluded from the first adherence trial because his small size limited our ability to collect blood twice a week. The blended drug delivery mixture, consisting of FTC (20 mg/kg), TAF (1.5 mg/kg), 600 mg of bitterness masking powder, 1–1.5 tablespoons of Nutella^®^, and 0.5–1.0 tablespoon of peanut butter, was scooped into a miniature ice cream cone (Joy Mini Cone). Drug delivery cones were prepared weekly, maintained at -20 °C, and allowed to thaw for approximately 20 minutes before administration. Prior to morning feeding, trainers would engage macaques with the PRT routine of sitting at the cage opening for food delivery. Macaques were observed for medication consumption and adherence was documented using medication diaries. Once the dose was consumed, juice was given as reinforcer and chaser.

Animals were anesthetized twice weekly for blood collection and FTC/TAF was given by oral gavage on these days to ensure that stable level of ARVs were maintained. Additionally, fasting is required prior to anesthetization, precluding cone administration before the morning feeding. Days of gavage were excluded from the oral adherence analysis. Due to poor adherence in Group B, doses were not administered on weekends and the trial was terminated one week early.

We next modified the drug delivery mixture described above by adding approximately 0.5 tablespoons of honey. We initiated a second 30-day adherence trial with four macaques; which included the three moderately successful animals from Group A (10–47, 27300 and 30622) and the 7^th^ subject from the initial PRT cohort (28025). Group B was excluded due to limited success in the first trial and the need for animals in other studies. Daily cone administration, PRT methodology and adherence observation were the same as above. Blood was collected once weekly, and on these days FTC/TAF was given by oral gavage and no adherence data was recorded.

Lastly, an extended 6-month adherence trial was conducted utilizing identical parameters and the same selected animals from the second trial. After a sudden drop in adherence following a splenic biopsy, numerous food contingencies for FTC/TAF delivery were tested including juices, sugar cookie and chocolate chip cookie dough. During suboptimal adherence, FTC and TAF were given twice weekly by oral gavage. FTC-only food contingencies were also tested.

### Analysis of FTC-TP and TFV-DP concentrations in PBMCs

PBMCs were isolated weekly from 8 cc of blood collected in BD Vacutainer^®^ CPT^™^ Cell Preparation Tubes. BioLegend RBC lysis buffer was used to lyse red blood cells to minimize interference with TFV-DP determinations. Cells counts and viability were determined using a Guava Cell Counter and Cytosoft data acquisition and analysis software (version 6.02; Millipore Billerica, MA). A total of 3 X 10^6^ viable cells were pelleted, washed with 1 ml of physiological saline and resuspended in 500 μl ice-cold 80% methanol. Samples were vortexed for 1 minute and immediately frozen at—70°C. At the end of the study, all samples were pelleted to remove cellular debris and supernatants were transferred to new tubes.

Supernatants were dried down to concentrate, reconstituted in 100 μl of 50 mM ammonium acetate buffer (pH 7.0), and centrifuged at 17,000 × *g* to remove insoluble particulates. Intracellular TFV-DP and FTC-TP concentrations were measured using an automated on-line weak anion-exchange (WAX) solid-phase extraction (SPE) method coupled with ion-pair (IP) chromatography-tandem mass spectrometry (MS-MS) using previously published methods. TFV-DP and FTC-TP were monitored through 448→176, 488→230, 495→136, and 468→112 *m/z* fragments, respectively, with [^13^C_5_]adenine-labeled internal standards for each analyte. Calibration curves were generated from standards of TFV-DP and FTC-TP by serial dilutions in 80% methanol over the range from 0.25 to 10 nM. The lower limit of quantification is 10 ng/ml for TFV-DP and 25 ng/ml for FTC-TP. All calibration curves had *r*^2^ values of greater than 0.99 [9, 27, 28].

### Measurement of plasma cortisol levels

Cortisol levels in plasma were measured at Assay Services (a core lab of the Wisconsin National Primate Research Center) using a commercially available coated-tube radioimmunoassay assay kit (MP Biomedicals). Intra-assay variability ranged from 4.9–8.4%.

## Results

### Training of macaques

PRT parameters were defined in a cohort of seven male rhesus macaques who had no record, nor demonstrated any evidence, of prior training. Verbal commands were used to cue desired behavior, and correct responses received positive reinforcement, which included primary (grapes and juice) and secondary reinforcers (clicker and verbal praise). Training included two approximation steps; i) shifting location in enclosure so that reinforcers were easily administered by trainer and ii) sitting in the specified location. Within one month, all macaques were successfully trained to shift to a specific location in their cage and sit on cue.

**Fig 1:**
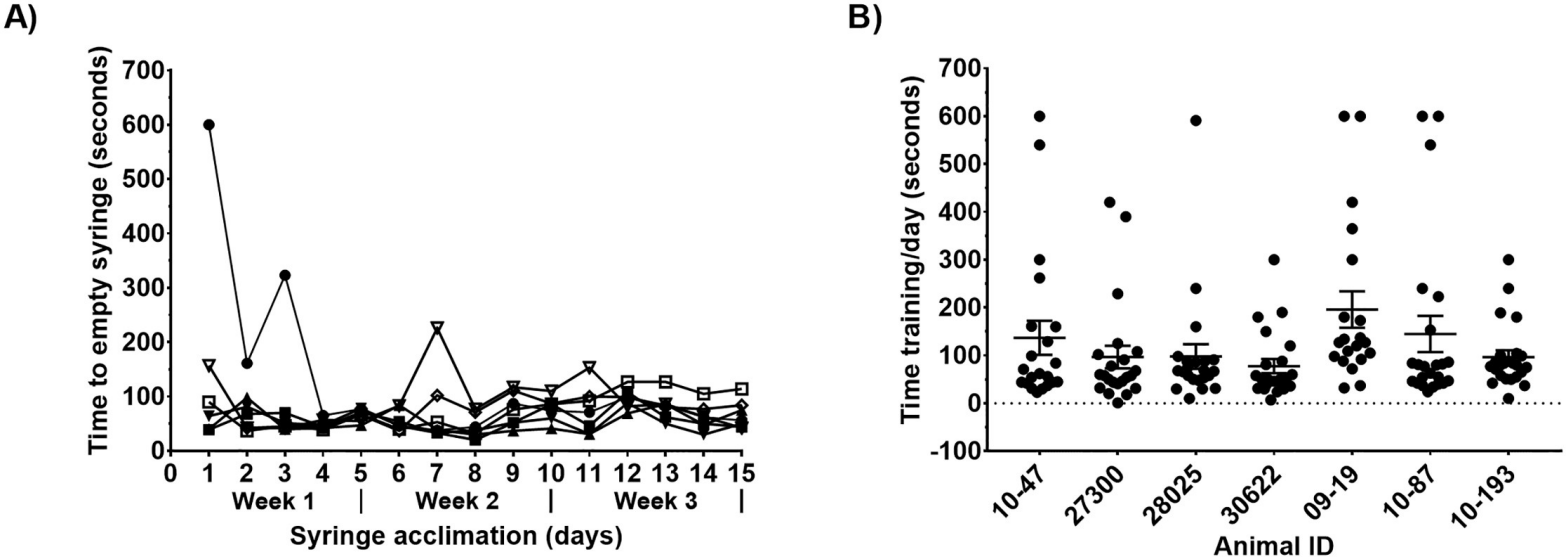
Positive reinforcement training. **A) Acclimation to syringe for juice training**. Male rhesus macaques (n = 7) were enrolled in a PRT program which used sweet liquids administered from a 60-cc catheter tip syringe as a reinforcer. Syringe acclimation was done over a period of three weeks, with training five days a week. Liquids tested included fruit juices (mango, apple, cranberry, passion fruit, pomegranate, and peach), diluted strawberry syrup, diluted molasses, vanilla ensure, kefir, yogurt and chocolate milk. Each individual animal is represented by a distinct shape/fill. **B) Daily positive reinforcement training (PRT) time**. Animal and trainer interactions were timed during training sessions with a stopwatch. Times when the animals did not successfully complete any desired behavior are included. Data shows results obtained over a two-month period when animals were trained to shift location in their cage, sit and accept liquid from a syringe. Individual daily training times are represented by circles; bars denote mean ± SEM.

The animals were acclimated to accepting liquid from a syringe, so it could be used as a delivery tool for medication or chasers. Integrating the syringe into PRT was done over a three-week period, with 15 days of total training. As shown in Fig 1A some macaques were initially hesitant to approach the syringe and drink the reinforcer. Observed behavior included fear responses such as grimacing, which were ignored and not reinforced. Animals quickly accepted liquid reinforcer and by the third week of syringe training the median time to empty 30 cc from the syringe was 74 seconds (range 20–600 seconds).

All seven macaques were successfully trained to shift location and sit in their enclosure and accept juice from a syringe within a two-month training period. Fig 1B illustrates the amount of time that was spent training each individual animal per day. Although some animals took to the training more quickly than others, a median of 67.5 seconds was spent training each animal daily.

### NRTI stability in food vehicles

Next, we investigated food delivery vehicles that successfully masked the taste of FTC and TAF. Examples of trial substances included fruit juices, yogurt, cookie dough, corn syrup, molasses, peanut butter, Nutella^®^, and honey. The macaques showed a preference for sweet fruit juices, peanut butter and Nutella^®^.

**Fig 2:**
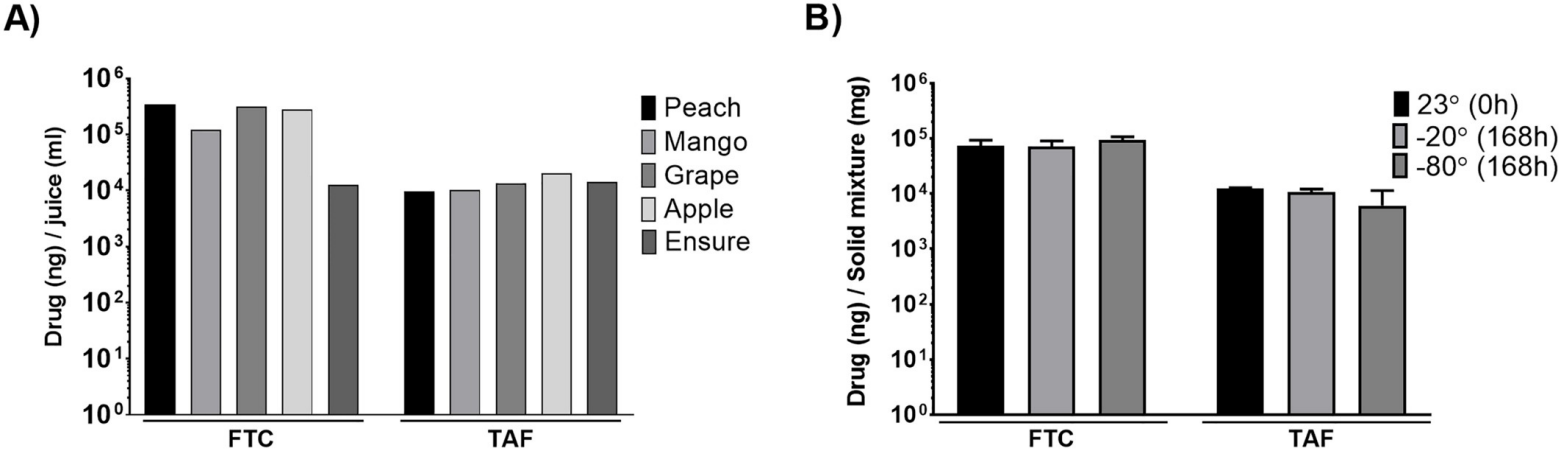
Drug stability in food. **A) FTC and TAF stability in liquids**. 200 mg of FTC, 15 mg of TAF, and 4% (w/v) natural bitterness masking powder (Fagron) were mixed into 30 ml of juice (peach, mango, grape, apple or Ensure) and vortexed until homogenous. Mixtures were tested for TAF and FTC concentrations by HPLC-MS/MS. Data is represented as ng of FTC or TAF per ml of liquid. **B) FTC and TAF stability in solid foods**. 200 mg of FTC, 15 mg of TAF, and 600 mg of bitterness masking powder were mixed well into 1 tablespoon of Nutella^®^ or 1 tablespoon of peanut butter (PB). This mixture was divided and maintained at -20°, or -80° C for one week prior to testing by HPLC-MS/MS. Control samples were made at room temperature (23°) and analyzed immediately. Data is represented as ng of FTC/TAF per mg of solid food (PB or Nutella^®^). Temperature and storage time had no effect on the concentrations of FTC or TAF measured by HPLC-MS/MS.

In order to define NRTI stability in the predetermined preferred foods, we considered the average weight of our cohort (10 kg) and used this weight to simulate TAF and FTC dosages. Human equivalent doses of TAF and FTC were combined with bitterness masking powder and mixed in 30 cc of juice until homogenous. Fig 2A shows the HPLC MS-MS analysis of FTC/TAF juice mixtures. TAF concentrations were similar in all vehicles. FTC concentrations were also similar with the exception of Ensure (Fig 2A).

Weighing individual doses and prepping drug mixtures daily is time consuming, and the alternative of thawing frozen juice mixtures at room temperature can take up to an hour. To improve logistics, we tested the stability of FTC/TAF prepared in solid food and stored at different temperatures for one week. FTC and TAF dosages for a 10 kg macaque were measured, combined with bitterness masking powder and mixed with Nutella^®^ or peanut butter (PB). Doses were kept at—20°C or—80°C for 7 days. Fig 2B shows the levels of FTC and TAF observed at each temperature with either Nutella^®^ or PB. Time and temperature had no impact on the stability of the drugs compared to the control of immediate preparation at room temperature. For the remainder of the study we chose a combination of PB/Nutella^®^ as our drug delivery method due to the ease of advance preparation and storage.

### Primary adherence trial showed limited success

Six trained macaques were chosen for a 30-day oral medication adherence trial and were arbitrarily housed into two separate rooms (Groups A, n = 3 and B, n = 3). Individual doses of FTC and TAF were weighed according to each animal’s weight, blended with bitterness masking powder, PB, Nutella^®^ and scooped into a miniature ice cream cone for ease of administration.

**Fig 3:**
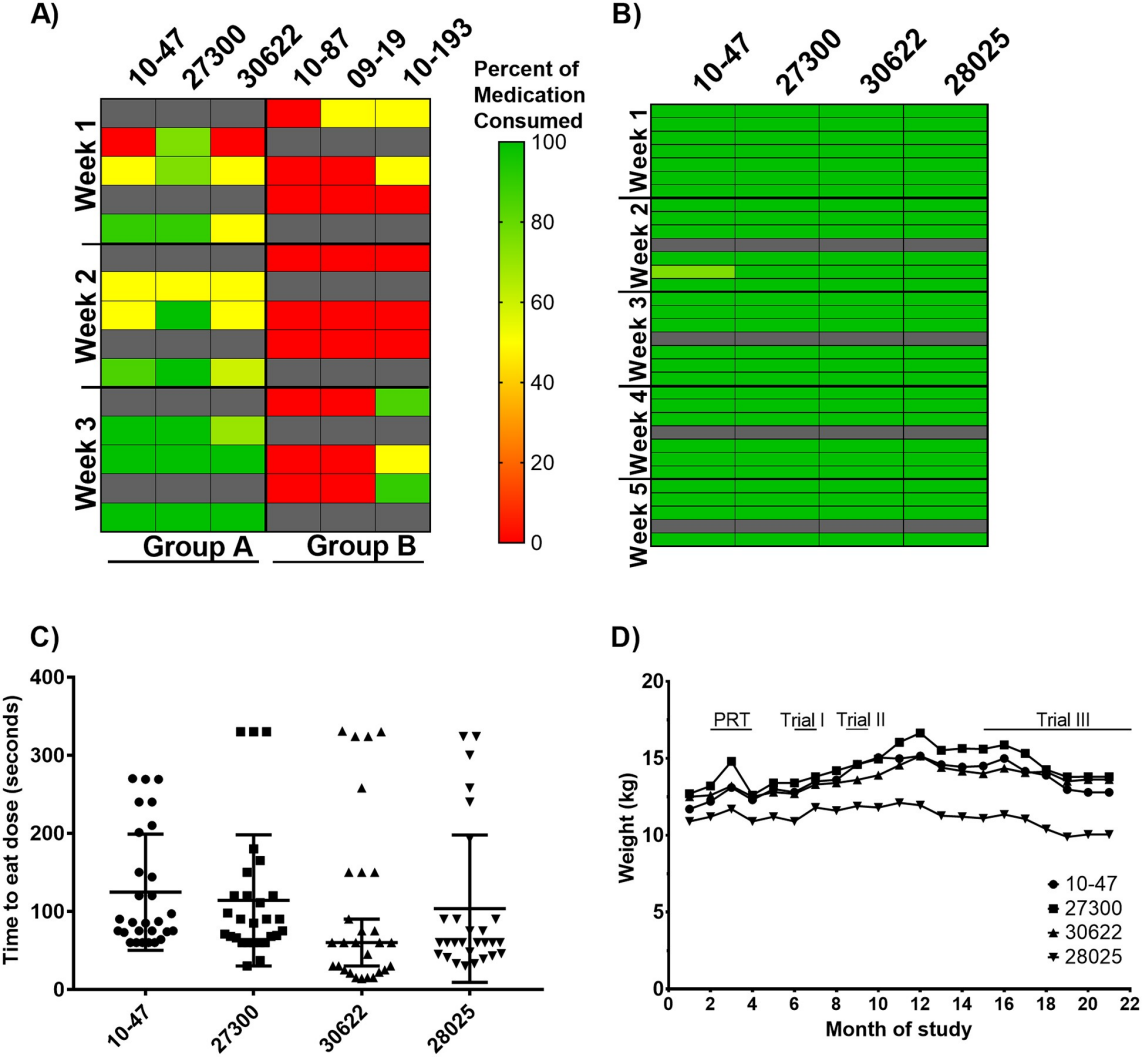
Adherence to daily oral FTC/TAF. **A) Drug adherence in trial I**. PRT trained macaques (n = 6) were enrolled in a 30-day oral FTC/TAF adherence trial with Groups A and B housed in separate rooms. Animals were observed for daily consumption of ARVs. Days of drug administration are denoted in rows (one box = one day); the six animals are represented by the columns. Colors in the heat map reflect 100% of the dose eaten (green), 50% of dose eaten (yellow), and 0% of dose eaten (red). Animals were anesthetized twice a week for blood collection; FTC/TAF was given by oral gavage on these two days (gray boxes). Doses were not administered on weekends.** B) Drug adherence in trial II**. PRT-trained macaques (n = 4) were chosen for a second 30-day adherence trial. The only deviation from the first trial was the addition of honey to the drug delivery mixture. All macaques were housed in the same room. Drugs in this trial were also given on weekends. **C) Dose consumption time during drug adherence trial II**. Individual daily times are represented by filled shapes and bars denote the mean ± SD time for each animal.** D) Changes in animal weight over the course of training**. Animal weights were monitored prior to training, during PRT and throughout adherence trials. Longitudinal timeline of training and adherence trials are indicated.

Prior to morning feeding, macaques were given the verbal prompts to sit in the specified cage location before administration of the drug delivery cone. Animals were observed for dose consumption. Juice was given as a chaser to encourage any remaining medication to be dislodged from the cheek pouch and as positive reinforcement for taking the cone. The adherence heat map in Fig 3A shows poor compliance during the first week with most macaques eating a partial dose or not eating the dose at all. By trainer observation, the mean dose consumed during the first week was 53% for Group A and 16% for Group B. Due to low adherence doses were not administered over the weekends. Behavior observed included hypersalivation, playing with the dose, eating the cone only and rubbing the mixture on the enclosure. Adherence at week 2 increased to 66% in Group A and was 0% in Group B. Adherence in Group A further increased to 96.6% at week 3 and remained low in Group B animals with the exception of macaque 10–193. Overall, only three of the six animals had greater than 50% adherence to the daily oral FTC/TAF regimen during this trial.

### Addition of sweetener improved adherence

Despite limited success of the first adherence trial, daily PRT was continued using cones without ARVs to ensure that animals retained trained behavior. We postulated that the poor adherence may have been due to medication bitterness and taste aversion. Therefore, we added honey to the blended drug delivery mixture (peanut butter, Nutella^®^, and bitterness masking powder) in an effort to mask the taste further. We initiated a second 30-day adherence trial and drug delivery cones were administered 6 days a week and oral gavage of FTC/TAF was given during the weekly blood collection. Fig 3B shows that the addition of honey resulted in 99.7% adherence in 4 macaques with 115/116 complete doses eaten.

One of the primary research concerns for oral drug administration in animal models is the input of time required for observation. During the second adherence trial observations, macaques were timed from dose administration to dose completion (Fig 3C). The median consumption time was 73 seconds [range 14–331 seconds], which is similar to the amount of time required to administer drugs via a subcutaneous injection. A secondary research concern is weight gain, a likely possibility considering macaques are eating a calorically dense treat every day. We did not find a significant weight change through 21 months of PRT and adherence trials, with a mean increase of 0.615 kg [range -0.85–1.12 kg] (Fig 3D).

**Fig 4:**
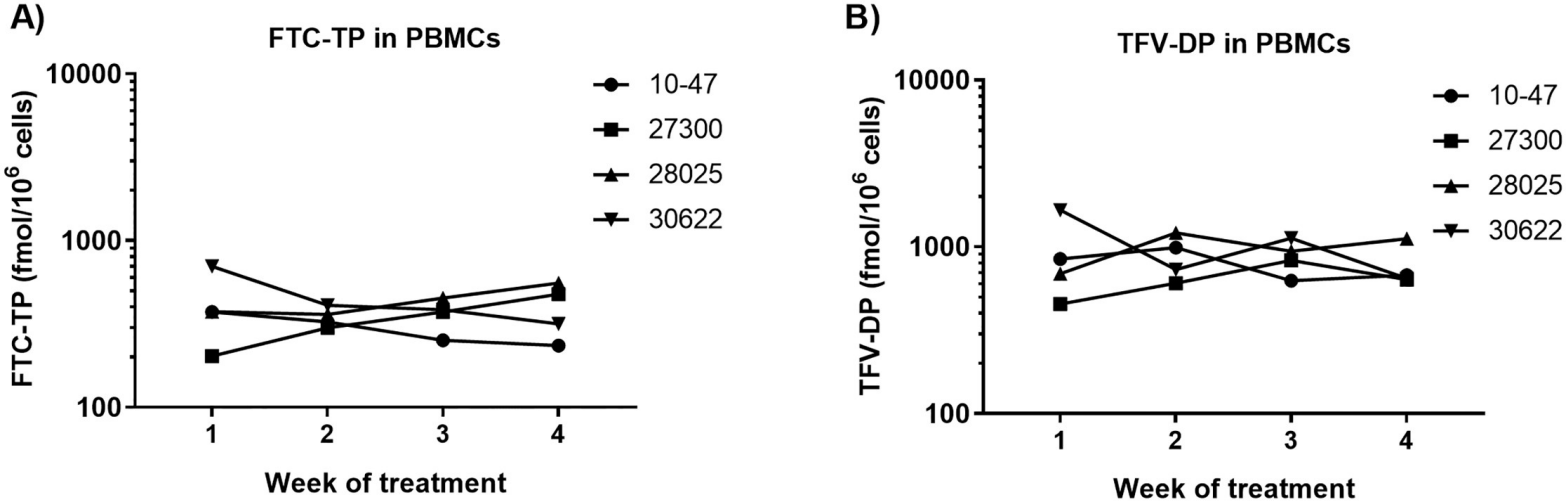
FTC-TP and TFV-DP concentrations in PBMCs. Data reflects the concentrations of FTC-TP (A) and TFV-DP (B) measured in PBMCs from 4 macaques treated with daily oral FTC/TAF.

As an objective measure of adherence, we collected blood weekly and quantified intracellular concentrations of TFV-DP and FTC-TP in PBMCs during the second trial. Levels of FTC-TP (median = 367.0 fmol/million cells [range, 289.5–413.5]) and TFV-DP (median = 845.8 fmol/million cells [range, 620.8–1031.3]) were stable and within the protective range achieved with daily dosing in humans (Fig 4A and 4B). These findings further support the observations of consistent dose consumption.

### Adherence during extended treatment with FTC/TAF

**Fig 5:**
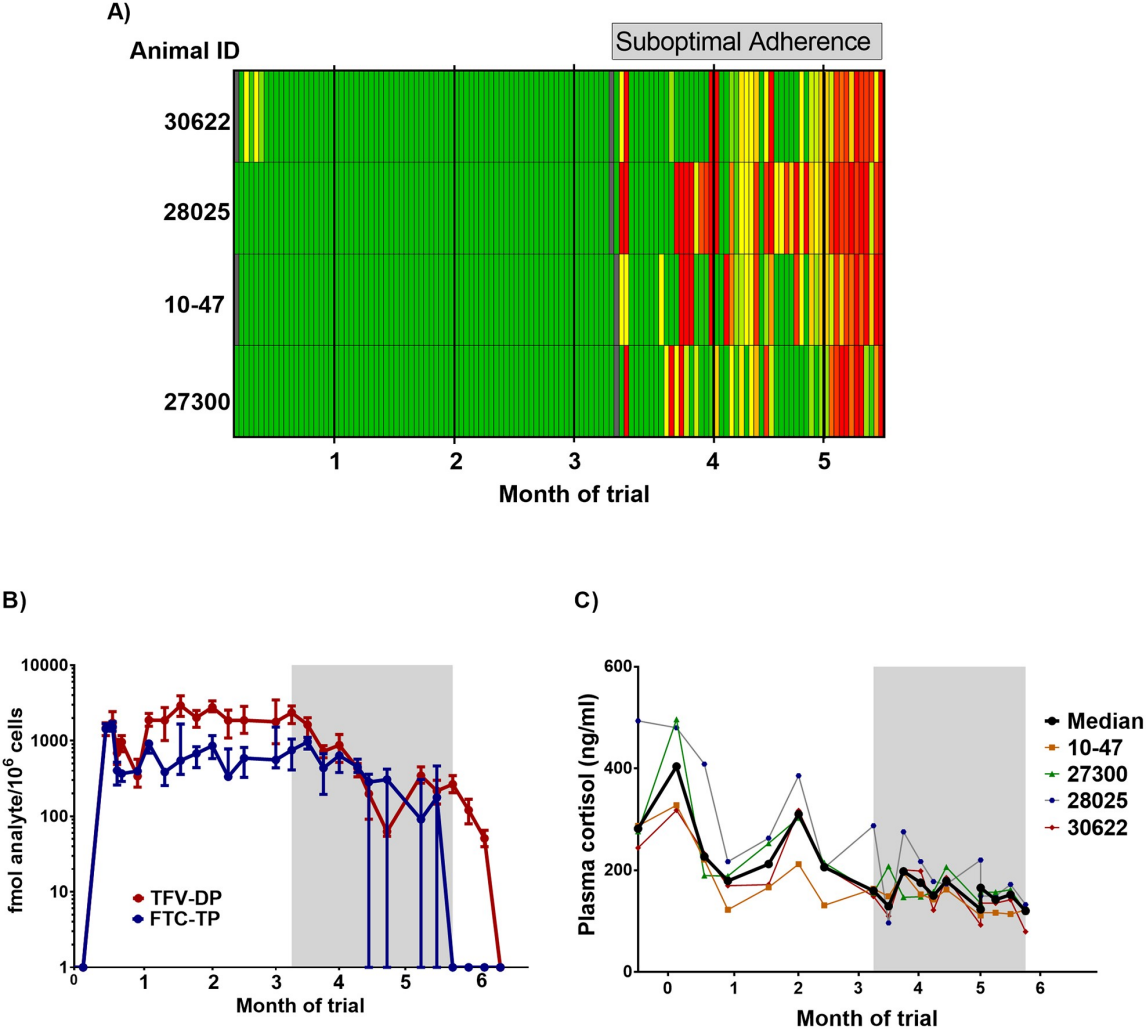
Adherence during extended treatment. **A) Adherence heat map**. Macaques (n = 4) were administered daily doses of FTC/TAF in a blended drug delivery mixture. Days of drug administration is denoted in columns (one box = one day) and the four animals in the rows. Colors in the heat map reflect 100% of the dose eaten (green), 50% of dose eaten (yellow), and 0% of dose eaten (red). Days of drug administration by gavage are not included in the adherence map. Day of spleen biopsy are indicated with gray boxes. **B) Concentrations of FTC-TP and TFV-DP in PBMCs**. FTC-TP and TFV-DP were stable during the first three months coinciding with high adherence to daily oral FTC/TAF. The period of suboptimal adherence is highlighted by gray bar. **C) Plasma Cortisol levels**. The gray area denotes the period of suboptimal adherence.

Next, we investigated the longevity of adherence to daily oral FTC/TAF. Fig 5A shows the adherence heat map during 5.5 months of treatment. Adherence was nearly perfect (99.5% or 352/356 doses consumed) during the first three months, with imperfect adherence only noted in one animal (30622) who ate 50–90% of the dose during the first week. However, adherence suddenly dropped soon after the macaques underwent surgery for a splenic biopsy. We attempted to regain compliance by utilizing our preplanned food contingencies to deliver FTC/TAF, including juices and cookie dough, but only observed sporadic success. Next, we removed TAF from the oral doses completely because of its extreme bitterness compared to FTC. Despite removing TAF, adherence to the FTC-only cones was limited for the remainder of the study.

The first three months of consistent medication consumption showed stable levels of FTC-TP and TFV-DP in PBMCs (Fig 5B). As expected, FTC-TP and TFV-DP concentrations during suboptimal adherence decreased. In an attempt to maintain protective TFV-DP and FTC-TP concentrations in PBMCs, we administered FTC/TAF twice a week by gavage. Twice weekly dosing by gavage and sporadic adherence to cones maintained detectable levels of FTC-TP and TFV-DP through the remainder of the study, although FTC was undetectable in some animals due to its short half-life. Fig 5B shows that TFV-DP and FTC-TP levels slowly declined during the period of suboptimal adherence and twice weekly gavage compared drug levels achieved with daily oral FTC/TAF. After cessation of treatment, FTC-TP was undetectable within one week and TFV-DP was undetectable within two weeks.

Psychological and physiological stress can induce an increase of serum cortisol. We investigated if stress related to the collection of splenic biopsies might have caused the sudden drop in adherence. Fig 5C shows no increase in plasma cortisol at the time of the splenic biopsy or in the weeks thereafter, when adherence was waning. In fact, cortisol levels decreased over the course of the study, indicating that long-term training may have contributed reduced stress in these animals.

## Discussion

We show that macaques can be trained to take daily oral ARVs using a combination of PRT, masking medication taste with high-value foods and the use of a juice chaser. We found that a small amount of training time yielded animals that sit in a specific cage location and accept juice from a syringe. Once we identified a food vehicle that masked the bitter taste of FTC and TAF, we were able to consistently administer blended oral doses that were consumed quickly. Near perfect medication adherence was observed for a 1-month trial and for the first three months of a long-term trial.

Improving macaque models of HIV to better reflect human HIV infection and treatment leads to increased translational relevance and informative preclinical data. Mimicking oral ARV administration in macaques has numerous challenges including time required to mask medication bitterness through trial and error, training animals for rapid medication consumption, and observing for dose consumption. Due to these logistical challenges, many macaque studies rely on subcutaneous injections or weekly/bi-weekly doses of drugs given by oral gavage under anesthesia [2, 16, 27, 29]. However, subcutaneous injections do not mimic the human route of administration and may alter drug biodistribution. Drug administration by oral gavage mimics the route of administration in humans but cannot be performed more than 1–2 times a week since it requires anesthesia and requires fasting precluding the ability to study the effect of food. In addition, infrequent dosing reduces the amount of drug and effective antiviral activity. This was evident in our study as we noted a decrease in FTC-TP and TFV-DP concentration during the period of suboptimal adherence when the macaques were receiving ARVs by gavage twice weekly.

FTC and TAF are available as FDA-approved single tablet formulations; TAF is recommended to be taken with food, while FTC can be taken without regard to food. These recommendations informed our decision to give FTC/TAF mixed into food for our trial. We noted that steady-state FTC-TP levels in PBMCs were lower than those achieved when a single dose of FTC is given orally by gavage without food. We surmise that the lower than expected FTC-TP concentrations might be due to the administration with a high fat meal which in humans caused a 29% decrease in plasma C_max_ for FTC [30].

Ensuring reliable oral medication consumption in macaques is fraught with many logistical challenges. Firstly, many medications including FTC and TAF have an extremely bitter taste. After limited success of our first adherence trial, we added honey to our blended drug delivery mixture. We found that the addition of honey increased adherence, although this finding could also be explained by subject selection since 3 of the 4 animals were already partially adherent during the first trial. More studies are needed to fully understand the role of sweeteners in masking bitter ARV medications. Secondly, macaque models often require regular specimen collection and anesthetization, which is known to induce appetite suppression. Interestingly, anesthesia once or twice weekly had no effect on adherence on the day immediately after anesthetization, potentially because the ‘high-value’ of peanut butter and Nutella^®^ superseded any anesthesia associated appetite suppression. Lastly, macaques have cheek pouches where foodstuffs can be held for later consumption or rejection, which can complicate the observation of dose consumption. Since liquids are not retained in this manner, we acclimated macaques to accepting juice delivered from a syringe which served three purposes: further masking of bitter taste with sweetness of the juice, encouraging purging of cheek pouches, and providing additional positive reinforcement for consuming medication. The combination of training, blending medication and a chaser may provide a template for other NHP studies requiring daily oral medication or other captive animal research. Importantly, this data may have other clinical implications as blending and chasing have been assessed in children with feeding disorders.

The time required for training is a main concern for oral medication delivery to animals. We trained macaques to sit and accept juice as positive reinforcement within 2 months. This timetable indicates that training can be done while animals are in quarantine or prior to initiation of any study. Our plasma cortisol data and other published research also indicate that training may contribute to reduced stress [31]. After PRT, macaques took their daily oral medication within ~70 seconds, which is similar to the amount of time needed to squeeze a cage and administer a subcutaneous injection. Oral administration by our methods is substantially less time consuming than anesthetizing and giving medication via oral gavage. Although there is an initial input of time and energy, these procedures are not complicated nor time consuming and yield animals that are more cooperative overall. We were successful in training all 7 animals, but only 4 consistently took the medication over several months. Future studies should evaluate other preferred foods or chasers that can further increase compliance.

Our study design included two commonly used HIV medications, FTC and TAF. The training parameters and food vehicles may require alterations for medications that vary in stability and bitterness. As we observed during our extended adherence trial, consistency of oral medication delivery is not guaranteed, and a variety of contingency food should pre-evaluated. We hypothesize that the sudden drop in adherence was due to conditioned taste aversion. The macaques were likely feeling unwell during their recovery from the splenic biopsy surgery. When macaques received their oral dose the subsequent day, an association with the taste of the drug delivery cone may have been made. Although we prepared alternate contingencies, we only had sporadic success with adherence. This might be due to the strong smell or taste of FTC, TAF or bitterness masking powder within the food vehicles. Because of this, we recommend that studies that use daily oral medication should limit surgically invasive procedures. However, less invasive specimen collection that require anesthetization such as blood draws, rectal wicks, rectal biopsies and lymph node biopsies had no effect on adherence in our trial.

In summary, we have refined macaque models of HIV treatment by providing a training protocol to administer daily oral ARV drugs. By mimicking the human route of drug administration and doses, our model has the potential to increase the translational relevance of preclinical studies of PrEP, PEP and treatment. The implications of this work go far beyond that of HIV and ARVs, as these techniques can be applied to studies of drug biodistribution and pharmacodynamics in nonhuman primate models, oral medication training of other captive animals and clinical research of feeding disorders.

## Supporting information

S1 Dataset Positive reinforcement training times. (XLSX)

S2 Dataset Drug stability in food. (XLSX)

S3 Dataset Adherence to daily oral FTC/TAF. (XLSX)

S4 Dataset FTC-TP and TFV-DP concentrations in PBMCs. (XLSX)

S5 Dataset Adherence during extended treatment. (XLSX)
